# Thermogel Delivers Oxaliplatin and Alendronate *in situ* for Synergistic Osteosarcoma Therapy

**DOI:** 10.3389/fbioe.2020.573962

**Published:** 2020-09-15

**Authors:** Yifu Sun, Ke Li, Chen Li, Ying Zhang, Duoyi Zhao

**Affiliations:** ^1^Department of Orthopedics, The Second Hospital of Jilin University, Changchun, China; ^2^Department of Orthopedics, The Fourth Affiliated Hospital of China Medical University, Shenyang, China; ^3^Department of Orthopedics, Zhongshan Hospital Affiliated to Xiamen University, Xiamen, China

**Keywords:** thermo-sensitive hydrogel, oxaliplatin, alendronate, immune activation, osteosarcoma therapy

## Abstract

The therapeutic effect of osteosarcoma (OS) has not made extraordinary progress in the past few decades. Oxaliplatin (OXA) is a widely used clinical anti-tumor drug. Recent studies have shown that OXA can trigger anti-tumor immunity by inducing immunogenic death (ICD). Alendronate (ALN) has been used to threaten the skeletal system tumors because of the unique bone affinity and the ability to inhibit bone destruction. In this study, we co-loaded OXA and ALN on mPEG45–PLV19 thermo-sensitive hydrogel to perform *in situ* treatment on the mouse OS model. Slowly released OXA can induce immunogenic death of tumor cells. At the same time, thermo-sensitive hydrogels can induce the accumulation of cytotoxic T lymphocytes. Besides, ALN could synergistically diminish tumors and prevent bone destruction. This system could synergistically inhibit the progression of OS and lung metastasis and has no toxicity to various organs throughout the body.

## Introduction

Osteosarcoma (OS), as the highest incidence of malignant bone tumor, has been harmful to human health (Wang et al., 2020). OS often occurs to adolescents (Tinkle et al., 2019), especially to boys. At present, the standard treatment of OS includes neoadjuvant chemotherapy, surgical resection, and post-operative chemotherapy (Zhang et al., 2020). Despite the deepening of research in this field, there has been no significant improvement in patient survival in recent decades (Harrison et al., 2018). Lung metastasis is an essential factor of the poor prognosis of OS (Huang X. et al., 2019).

In recent years, immunotherapy has provided new directions for the treatment of cancer. Researchers have found that some current chemotherapy drugs can induce immunogenic cell death (ICD) of tumor cells, and the mechanism is related to the induction to apoptosis, including the exposure of calreticulin (CRT) on the cell membrane (Sukkurwala et al., 2014). ICD would release tumor antigens, thereby triggering tumor immune response (Menger et al., 2012; Martins et al., 2014). Platinum drugs are the first-line chemotherapy drugs for the treatment of OS (Cheng et al., 2020). Oxaliplatin (OXA) is the third-generation platinum anti-tumor drug that target DNA as the site of action. Platinum atoms form a cross-link with DNA to antagonize its replication and transcription. Researchers have found that OXA could induce anti-tumor immunity, which produces anti-tumor immune effects by initiating ICD, acting on cellular STAT protein signaling pathway, and regulating the immunosuppression tumor microenvironment (Ghiringhelli et al., 2009; Hato et al., 2014). There is evidence that OS may be sensitive to immunotherapy. The percentage of infiltrating CD8^+^ T lymphocytes in OS was higher than that in other sarcoma subtypes (van Erp et al., 2017), and the degree of infiltration was positively correlated with survival rate of patients (Gomez-Brouchet et al., 2017). OS has a high level of genomic instability and expresses programmed cell death protein-1 ligand (PD-L1) (Koirala et al., 2016), indicating potential sensitivity to inhibitors of PD-1/PD-L1 pathway (Kansara et al., 2014; Mouw et al., 2017).

Bisphosphonate is a successfully bone resorption inhibitor, which has a strong affinity with hydroxyapatite in bone. It could inhibits the activity of osteoclasts, thus inhibits the bone destruction caused by OS (Blanco et al., 2009). Alendronate (ALN) is one of the most commonly used bisphosphates in the clinic (Uihlein and Leder, 2012). It is mainly used for the treatment of osteoporosis caused by menopause, glucocorticoid disorders, and parathyroid disease (Wang et al., 2017, 2018; Jiang et al., 2020). ALN has achieved satisfactory therapeutic effects in the above diseases.

Thermo-sensitive hydrogel is widely used in biological research (Wei et al., 2017; Liu et al., 2018; Wang C. et al., 2019; Wang Y. et al., 2019; Zhang et al., 2019). It has injectability, suitable gel-forming ability, excellent biocompatibility, degradability, and excellent drug loading capacity (Huang H. et al., 2019). Hydrogel delivery drugs have been extensively studied (Li et al., 2018; Zhang et al., 2018; Ning et al., 2019; Qiu et al., 2020; Zheng et al., 2020). Studies have confirmed that hydrogels are able to serve as an immune adjuvant to recruit immune cells after implantation (Wang Y. et al., 2019).

In this study, polyvaline (PLV) thermo-sensitive hydrogel loaded with OXA and ALN was used for the treatment of OS. We aim to induce the ICD of OS cells through the release of OXA in the tumor site, thereby promoting the tumor immune response. At the same time, PLV hydrogel can recruit immune cells to synergistically enhance the immunotherapeutic effect. In addition, ALN could synergistically treat tumors and protect bone tissue against the damage caused by tumor invasion.

## Materials and Methods

### Cells and Animals

The mouse OS cell line K7M2 is routinely cultured in DMEM medium (Invitrogen) containing 10% neonatal bovine serum (Beijing Solabe Technology Co., Ltd.), and the cells are collected until they grow to about 80%. SPF grade BALB/c female mice (4 weeks) were purchased from Beijing Weitong Lihua Experimental Animal Technology Co., Ltd. for use *in vivo* anti-tumor experiments of PLV hydrogel. All mice were kept in a clean and pathogen-free environment, the temperature was maintained at 20–22°C, and the light-dark cycle was 12 h. All mice were used and handled according to the protocol approved by the Jilin University Animal Protection and Use Committee.

### Thermo-Sensitive Hydrogel Loaded With Drugs

First, the terminally aminated polyethylene glycol monomethyl ether is prepared by the hydroxyl amination reaction of polyethylene glycol monomethyl ether. Take 30 g of mPEG, dissolve it in 300 mL of dichloromethane, add 8.4 g of potassium hydroxide, and 14.17 g of p-toluenesulfonyl chloride, and stir them at room temperature for 7 days. After the completion of the reaction, the dichloromethane solution was collected in a separatory funnel and washed with saturated sodium chloride aqueous solution six times, each about 30 mL. Collect the lower methylene chloride layer, add anhydrous magnesium sulfate, and dry overnight. The dried dichloromethane solution was suction filtered to remove magnesium sulfate. The resulting solution was concentrated to 100 mL by rotary evaporation, and then settled with 10 times volume of ice ether for three times. The intermediate product was collected by filtration through a Buchner funnel and then drained by a cold trap. Weigh the mass of the intermediate product, add the equal mass of NH_4_Cl, and add 30 times the volume of ammonia to dissolve it, and stir for 7 days. Collect the solution in a separatory funnel, add sodium chloride until the ammonia solution is saturated, add dichloromethane for extraction, add about 60.0 mL each time, extract five times, collect the lower liquid, add anhydrous magnesium sulfate and dry overnight. Filtered with a sand core funnel, the product was concentrated to about 100.0 mL, 10 times the volume of ice ether was settled three times, and the terminally aminated mPEG_2000_-NH_2_ was obtained after suction.

After that, L-Val NCA was prepared by triphosgene and L-valine in the tetrahydrofuran solution. Add 400 mL of tetrahydrofuran to a dried three-necked flask, then add 15 g of L-valine and 9 g of triphosgene, slowly ventilate nitrogen and place the device in a 60°C oil bath and stir for about 1 h. After the solution is clear, add 15 g of L-valine and 9 g of triphosgene again, and continue the reaction. After about 2 h, increase the nitrogen flow rate, blow the solution to about 100 mL, and settle the reaction solution with a 10-fold volume of n-hexane solution at a temperature of −20°C. After the sedimentation is complete, filter the resulting product with a funnel, collect the upper filter residue, and dissolve it with 200 mL of ethyl acetate at a temperature of −20°C. Collect the ethyl acetate solution in a separatory funnel, wash the solution with a saturated sodium bicarbonate solution at 4°C, 30 mL each time, wash six times, and collect the supernatant. Then wash the solution with 4°C water, 30.0 mL each wash six times, and collect the upper layer. Collect the washed ethyl acetate solution into an Erlenmeyer flask, add an appropriate amount of anhydrous magnesium sulfate, and place it in a refrigerator at –20°C for about 4 h.

The crude L-Val NCA was recrystallized. Filter the dried ethyl acetate solution with a sand core funnel into the dried bottle. After collecting the solution, connect the cold trap to drain the ethyl acetate to obtain crude L-Val NCA. The drained product was dissolved with 30 mL of tetrahydrofuran at 40°C, and then 10 mL of n-hexane solution was added until precipitation precipitated, and then dissolved at 60°C. Subsequently, the recrystallized L-Val NCA solution was placed in a refrigerator at –20°C overnight. The recrystallized L-Val NCA upper layer solution is drawn out and then drained by a cold trap to obtain refined L-Val NCA.

Finally, mPEG_45_-NH2 was used as a macroinitiator to initiate the ring-opening polymerization of L-valine NCA to obtain mPEG_45_-PLV_19_. Dissolve 2.86 g of L-Val NCA in 100 mL of DMF. Reacted at room temperature for 3 days, and then settled with 1000 mL of ice ether. The obtained product was dissolved in 40 mL of DMF and placed in a dialysis bag with a molecular weight cut-off of 5000 Da for dialysis for 3 days. After freeze-drying, the final product mPEG_45_-PLV_19_ was obtained.

mPEG_45_–PLV_19_ (5.0 wt%) was stirred slowly and thoroughly at 4°C overnight, and OXA (5.0 mg/mL) (Feather et al., 2018) and ALN (10.0 mg/mL) (Shan et al., 2020) were added to the polymer solution and mixed thoroughly (4°C) to keep injectable. The sol state is used for tumor suppression experiments.

### Degradation and Drug Release *in vitro*

To simulate the degradation of the gel in the body, we chose a buffer solution prepared from PBS, elastase (0.2 g/L), calcium chloride (CaCl2, 10.0 mmol/L), and sodium azide (NaN_3_, 0.2 wt%) as the degradation medium. Add 3 mL of buffer solution to the top of the gel. Change the buffer solution every 3 days and accurately weigh the remaining gel to measure the biodegradation rate.

Place the mPEG_45_–PLV_19_ (5.0 wt%) gel in a glass bottle and cover the gel with 3 mL PBS. Place the glass bottle in a constant temperature shaker at 37°C. The PBS solution was changed every 3 days. Detect the release of OXA and ALN by elemental analysis (ICP).

### MTT Assay

Transfer 100 μL of K7M2 in the medium to a 96-well plate (5000 cells/well). After culturing for 24 h, add 100 μL of medium or mPEG_45_–PLV_19_ (5.0 wt%) Gel. After treatment for 0, 24, and 48 h, add 20 μL of tetramethylazolium salt indicator (5 mg/mL in PBS, pH 7.4) to each well, and incubate for another 4 h in the dark at 37°C. Measure the absorbance of the solution at 490 nm on a microplate reader. Determine the relative survival rate of cells by comparing the absorbance of different groups. The data are expressed as mean ± SD.

### Establishment of Osteosarcoma Model and Animal Grouping

K7M2 cells in the logarithmic growth phase were digested with trypsin, washed three times in pre-chilled PBS, and suspended in cold PBS, ready for the preparation of OS *in situ* model. Anesthetize the mouse and remove the right lower limb hairs to fully expose the front surface of the right tibia and knee joint. Flex the mouse’s knee at a 90° position, using a 1 mL syringe needle, slowly rotate to vertically penetrate the cortex of the right tibia, and insert the needle along the long axis of the tibia until it is inserted into the metaphysis of the tibia about 3.5 mm. Sampler, inject 25.0 μL of cell suspension (2 × 10^6^ K7M2 cells) into the medullary cavity of the tibia. After the injection is completed, a certain pressure is applied to the injection site to prevent excessive pressure from causing the cells to ooze out. Monitor the tumor growth of the right leg of the mouse, and calculate and record the tumor region volume (V) using Eq. 1 (Li et al., 2020):

(1) $$V\left({\mathrm{mm}}^{3}\right)=\frac{4\pi }{3}\text{×}\frac{L}{2}\times {\left(\frac{AP}{2}\right)}^{2}$$

*AP* is the maximum length of the lateral diameter of the tumor measured along the horizontal axis; *L* is the maximum longitudinal length of the tumor measured along the long axis of the tibia.

When the tumor region volume reached 500 mm^3^, OS model mice were grouped. The mice were randomly divided into five groups, namely control group, OXA + ALN group, Gel + OXA group, Gel + ALN group, and Gel + OXA + ALN group, four mice in each group. The doses of OXA and ALN are 5 and 10 mg/mL, respectively, and 100 μL of 5.0 wt% PLV thermosensitive sol (at 4°C) evenly mixed with OXA or (and) ALN was injected slowly into the orthotopic OS tumor of mice Instead of the injection of mPEG_45_–PLV_19_ gel in the same amount of PBS, the control group was injected with the same amount of PBS. When the experiment ended (16 days), the mice were euthanized. All mice were processed according to the protocol approved by the Animal Protection and Use Committee of Jilin University.

### Data Collection of Evaluation Indexes for *in situ* Inhibition of Osteosarcoma

From the 0th day of administration, the body weight and tumor volume of mice were recorded at the same time every day, and the tumor inhibition curve, as well as tumor inhibition rate curve, were drawn. The tumor volume is calculated according to Eq. 1. On the 16th day of the experiment, the mice were sacrificed, and the hind limbs of the tumor were photographed and weighed, and then micro-CT scans were taken.

### Micro-CT

Scan the right calf specimens of tumor-bearing mice with micro-CT (micro-CT; Bruker, Skycan1172, Germany) and use computer software (Brook, Germany) for three-dimensional reconstruction to observe the bone destruction and further locate the tumor center Projection of the site on the anterior side of the tibia, using this area as the region of interest (ROI) for quantitative analysis of relative bone volume (BV/TV).

### Flow Cytometry

Take the tumor-draining lymph node and grind with the rubber end of the syringe to obtain a single-cell suspension. Tumor tissue was obtained, the tumor was minced with a scalpel, and treated with 1 mg/mL collagenase I (Gibco, United States) for 1 h. The cells were filtered through a nylon mesh filter and washed with PBS. The single-cell suspension was blocked with anti-CD16/32 (Invitrogen) on ice for 20 min to reduce non-specific binding to the immunoglobulin Fc receptor. The cells were further stained with antibodies conjugated to the following fluorescent dyes: CD45, CD3e, CD4, CD8 (all from Invitrogen). Flow cytometry was performed on LSRII (BD Biosciences), and data analysis was performed using FlowJo software (TreeStar).

### Pathological Analysis

On the 16th day after orthotopic injection of mPEG_45_–PLV_19_ gel, all mice were euthanized. The main organs and tumors were collected, paraformaldehyde (4.0%) was used to fix the main organs and tumor tissues, hematoxylin and eosin (H&E) were used to stain the tumors and organ sections, and the microscope (Nikon Eclipse Ti, Ardmore, PA) observes the changes in histomorphology.

## Results and Discussion

### Degradation, Drug Release, and Cytotoxicity of the Gel *in vitro*

**FIGURE 1:**
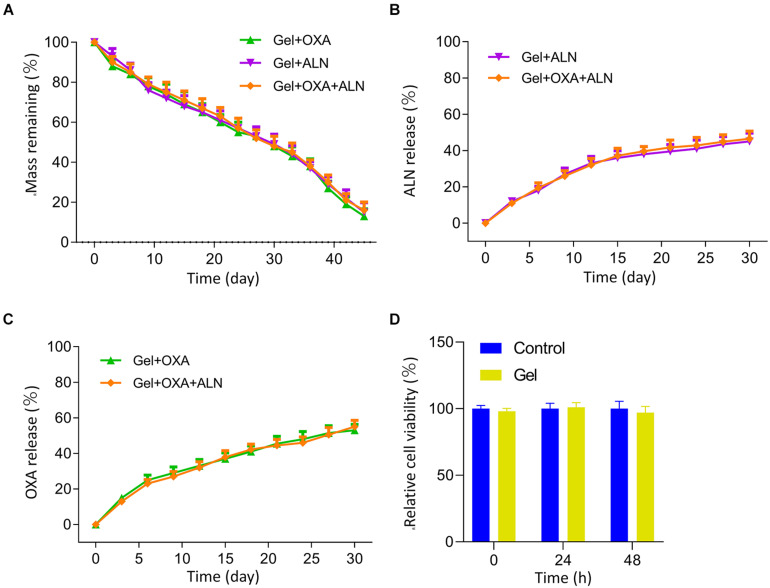
**(A)** Mass remaining of each group Gel *in vitro*. Cumulative release curves of ALN **(B)** and OXA **(C)** from each group *in vitro*. **(D)** MTT assay was used to measure the effects of the Gel on viabilities of K7M2 cells at 0, 24, 48 h after dispose, the data is expressed as mean ± SD (*n* = 3).

We analyzed the *in vitro* degradation properties of the gel. The result is shown in Figure 1A. We found that all three groups of gels exhibited similar degradation rates in the buffer solution of simulated body fluids. The degradation process was stable, and there was no rapid weight loss at the initial stage of degradation. And the degradation rate reached 50% in about 27 days.

The results of drug release *in vitro* are shown in Figures 1B,C. The drug-loaded in gel achieves a sustained-release effect. None of the three groups of drug-loaded gels showed the rapid release of early drugs. The gel loaded with OXA and/or ALN slowly released about 40% of the drug within 15 days.

We verified the effect of gel on K7M2 cell activity through MTT assay. The result is shown in Figure 1D. We measured the cell activity at 0, 24, and 48 h, and the results showed that the existence of gel does not affect the activity of tumor cells.

The above experiments show that mPEG_45_–PLV_19_ temperature-sensitive gel is an ideal drug-loaded gel. It has an appropriate degradation rate and drug release rate and can achieve sustained release of drugs *in situ*, which is conducive to maintaining local drug concentration to achieve the ideal therapeutic effect.

### Evaluation of Thermosensitive Hydrogel Synergistic Delivery of Oxaliplatin and Alendronate Anti-tumor Effects *in vivo*

**FIGURE 2:**
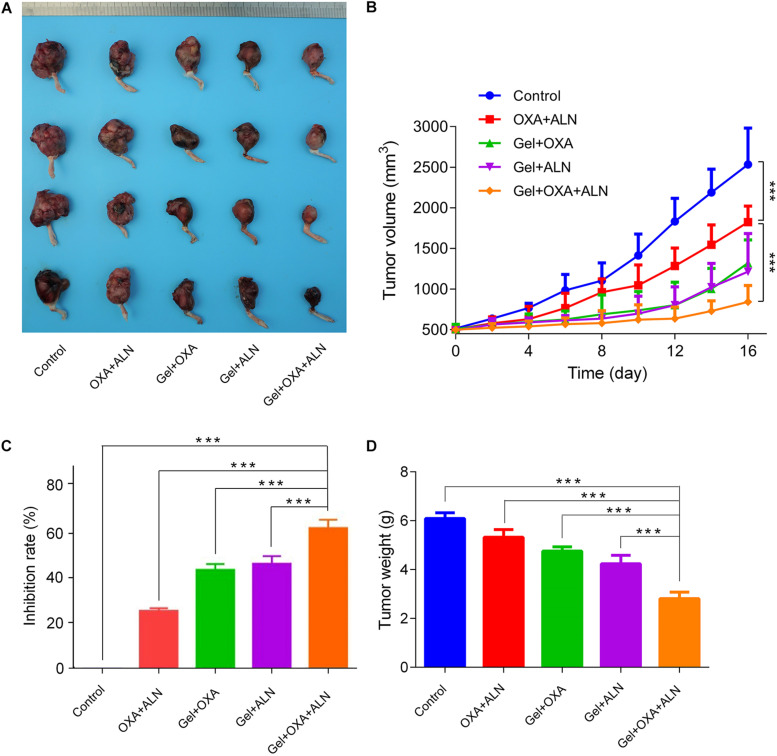
**(A)** Tumor pictures after treatment in each group. The inhibition curve **(B)**, tumor inhibition rate **(C)**, and the weight of osteosarcoma after treatment **(D)** of each group, the data is expressed as mean ± SD (*n* = 4; ***P < 0.001).

After the experiment, the mice were euthanized. The osteosarcoma tissue *in situ* was excised and photographed. As shown in Figure 2A, the tumor volume progression of each treatment group was slower than that of the blank group. Among them, the OXA + ALN group showed an unfortunate tumor-suppressive effect than other three treatment groups, which indicates that it is necessary to use the gel to achieve local sustained-release drugs. A single local injection of free drugs will not achieve satisfactory therapeutic effects due to faster metabolism. In the Gel + OXA + ALN group, The growth rate of tumor volume achieved the most obvious slowed down, which suggested that the gel targeted delivery of OXA and ALN had a significant effect on inhibiting the formation of OS.

The tumor volume was measured as shown in Figure 2B. On the first day, the initial volume of each group was 514.4 ± 48.1 mm^3^ (Control group), 504.2 ± 56.9 mm^3^ (OXA + ALN group), 507.1 ± 59.6 mm^3^ (Gel + OXA group), 491.6 ± 45.2 mm^3^ (Gel + ALN group), and 499.6 ± 33.5 mm^3^ (Gel + OXA + ALN group), the tumor volume baseline was the same. At half of the experiment, on the 8th day after treatment, the tumor volume of each group was 1102.5 ± 220.3 mm^3^ (Control group), 962.1 ± 162.7 mm^3^ (OXA + ALN group), 689.2 ± 238.2 mm^3^ (Gel + OXA group), 635.1 ± 93.1 mm^3^ (Gel + ALN group) and 580.2 ± 153.9 mm^3^ (Gel + OXA + ALN group). In 8 days, the tumor volume of Control group mice increased rapidly, and the volume increase was as high as 3139.3 mm^3^. After the gel was loaded with OXA or ALN alone, the tumor was effectively controlled. In the Gel + OXA + ALN group, the tumor-suppressing effect was the best. At the end of the experiment, on the 16th day after treatment, the tumor volume in the Gel + OXA + ALN group was 841.2 ± 202.9 mm^3^, which was significantly smaller than that in the Gel + ALN group 1214.7 ± 468.5 mm^3^, in the Gel + OXA group 1320.6 ± 285.1 mm^3^, and OXA + ALN group 1824.8 ± 197.5 mm^3^. The tumor volume of the untreated control group was 2533.3 ± 448.1 mm^3^.

As shown in Figure 2C, there was a significant difference in the tumor inhibition rate among each group. The tumor inhibition rate of the OXA + ALN group was only 25.2%, and the tumor inhibition rates of the gel loaded with OXA and ALN alone were 42.0% and 43.8%, respectively. The Gel + MTX + ALN group had a tumor suppression rate of 60.4%, achieving the most significant tumor inhibition effect. The results indicated that the sustained release of gel and the combined use of synergistic drugs could maximize the therapeutic impact of *in situ* tumors.

The dissected tumor is weighed. As shown in Figure 2D, compared with other groups, the average weight of hind limbs with tumors in the Gel + OXA + ALN group was the smallest (2.81 ± 0.26 g). OXA + ALN group (5.32 ± 0.31 g) and control group (6.09 ± 0.23 g) tumor-containing hindlimb weights were close, and the results were consistent with tumor volume changes.

### Evaluation of Anti-bone Destruction Effects

**FIGURE 3:**
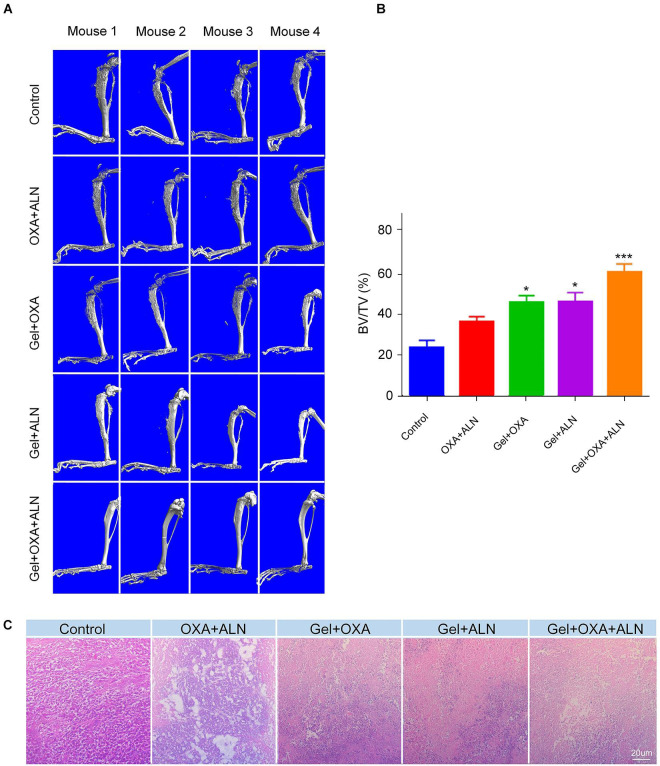
**(A)** Micro-CT images of orthotopic osteosarcoma after treatment in each group. **(B)** Relative bone volume of tibia of each treatment group, the data is expressed as mean ± SD (*n* = 4. Compared with Control group, *P < 0.05, ***P < 0.001). **(C)** Orthotopic tumor histopathology images after treatment in each group.

To further confirm the anti-tumor efficacy of Gel + OXA + ALN, the degree of bone destruction caused by local OS invasion was evaluated by micro-CT. As shown in Figure 3A, severe pathological bone destruction induced by OS progression was found in the proximal tibia of the control group mice without any treatment. The OXA + ALN group also had serious pathological bone destruction. In the Gel + OXA group, Gel + ALN group, and Gel + OXA + ALN group, the proximal tibia bone destruction of the lower limbs of the mice was weakened, and the degree of bone destruction in the Gel + OXA + ALN group was the most limited. The results of micro-CT indicate that the Gel + OXA + ALN group has the best protective effect on OS bone destruction. Similarly, as shown in Figure 3B, the relative bone volume (BV/TV) values of each group were compared: Control group (21.86% ± 2.87%), OXA + ALN group (34.22% ± 1.92%), Gel + OXA group (43.09% ± 2.38%), Gel + ALN group (43.51% ± 3.33%) and Gel + OXA + ALN group (56.61% ± 3.17%). We can also come to a similar conclusion that the degree of bone destruction in Gel + OXA + ALN group was the most limited, indicating the Gel + OXA + ALN provided the most potent protective effect on bone destruction.

In order to explain the reasons for the protection of bone tissue by this system, we further explored the inhibitory effect of the system on osteosarcoma tissue. OS *in situ* of mice was fixed with formaldehyde, then decalcified, embedded, sectioned, and finally stained by H&E. After that, the histopathological analysis was performed. The results are shown in Figure 3C. No necrosis-related symptoms were seen in the control group. *In situ* OS tissues of mice in the OXA + ALN group, Gel + OXA group, Gel + ALN group all had different degrees of necrosis. In the Gel + OXA + ALN group, severe cell atrophy, reduced density, nucleocytoplasmic concentration, and even tumor cell disappearance were observed. A large area of tumor cell apoptosis and necrosis were also showed. Therefore, the histopathological analysis further confirmed the anti-tumor efficacy and the potential of Gel + OXA + ALN for *in situ* anti-tumor treatment.

The results of tumor volume, weight, H&E staining, and pathological bone destruction related to the progression of the proximal tibia of the lower extremity and OS emphatically demonstrated the anti-tumor efficacy of Gel + OXA + ALN and the ability of inhibiting the bone destruction caused by OS.

### Evaluation of Anti-tumor Immune Effects

**FIGURE 4:**
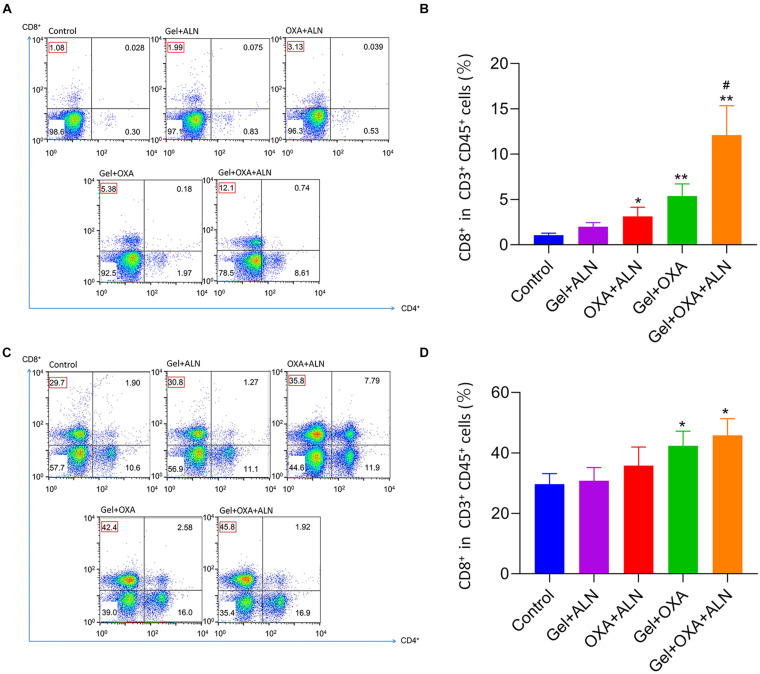
*In situ* OS were collected from mice 16 days after treatment. Representative flow cytometry analysis images **(A)** and the relative quantification gating on CD8^+^ cells in CD3^+^ CD45^+^ cells **(B)**. Data are presented as mean ± SD (*n* = 3). Lymph nodes adjacent to the tumor were collected from mice 16 days after treatment. Representative flow cytometry analysis images **(C)** and the relative quantification gating on CD8^+^ cells in CD3^+^ CD45^+^ cells **(D)**. Data are presented as mean ± SD (*n* = 3). Compared with Control group, *P < 0.05, **P < 0.01. Compared with Gel + ALN group, ^#^*P* < 0.05.

Tumor cells and lymphocytes of mice were collected for flow cytometry experiments. The anti-tumor immune effect caused by OXA was clarified by analyzing lymphocytes. After the treatment, the tumor tissues were isolated under sterile conditions and digested with collagenase to prepare a single tumor cell suspension. Flow cytometry was performed by antibody labeling, and CD3^+^ and CD45^+^, double-positive cells were analyzed. Representative flow cytometry analysis images were shown in Figure 4A, the CD8^+^ T cell infiltration rate of the tumor in the control group was 1.08%. The rate of CD8^+^ T cell infiltration induced by Gel + ALN injection near the tumor was 1.99%, no significant difference was observed with the control group. However, the injection rate of free OXA + ALN beside the tumor-induced the infiltration rate of CD8^+^ T cells to be 3.31%, confirming that OXA could induce ICD and increase the number of CD8^+^ T lymphocytes in the tumor. However, since a single local injection of free drugs will be rapid metabolized, free OXA + ALN group has not achieved satisfactory therapeutic effects. Gel + OXA induced CD8^+^ T cell infiltration rate to 5.38%, indicating that the gel can slowly release OXA, causing the sustained anti-tumor immune effect. The Gel + OXA + ALN group induced a CD8^+^ T cell infiltration rate of 12.1%, which was higher than any other treatment group. The reason may be that ALN has an inhibitory effect on the growth of osteosarcoma, which can indirectly increase the proportion of lymphocytes in the tumor and reduce the tumor’s resistance to immune cells. So that OXA and ALN have a synergistic therapeutic effect. We performed the above tests on three mice in each group, and the results were shown in Figure 4B. We performed a statistical analysis of the results and verified the above analysis results again.

In order to further verify the immune effect of the treatment, we collected tumor-draining lymph node lymphocytes for flow cytometry analysis. As shown in Figure 4C, CD3^+^ and CD45^+^ double-positive cells were used as analysis objects. The Gel + OXA + ALN treatment group had the highest CD8^+^ T cell infiltration rate (45.8%), higher than the Gel + OXA group (42.4%), free OXA + ALN group (35.8%), and Gel + ALN group (30.8%) and control group (29.7%). The results are consistent with the infiltration of CD8^+^ T cells in tumor cells, which proves once again that OXA induces ICD in tumor cells, stimulates the body’s anti-tumor immune response and achieves the goal of inhibiting OS progression. We performed the above tests on three mice in each group, and the results were shown in Figure 4D. We performed a statistical analysis on the results and verified the above analysis results again.

### Evaluation of the Inhibiting Lung Metastasis Effects

**FIGURE 5:**
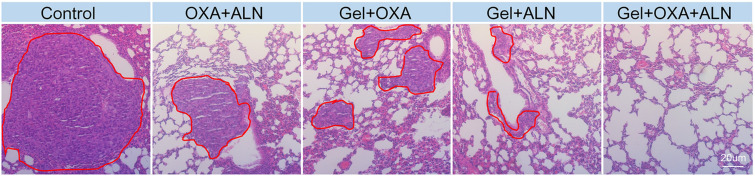
H&E images of lungs tissue in different treatment groups (Lungs metastases are marked in the red circle).

OS is often accompanied by lung metastasis, which significantly affects the prognosis of patients in clinic. The lung tissues were fixed, sectioned, and H&E stained for pathological analysis. As shown in Figure 5, the number and area of tumors in the lung sections of the Gel + OXA + ALN group were the smallest, showing that Gel + OXA + ALN had a more profound inhibitory effect on OS lung metastasis than other treatment groups.

### Evaluation of the Safety *in vivo*

**FIGURE 6:**
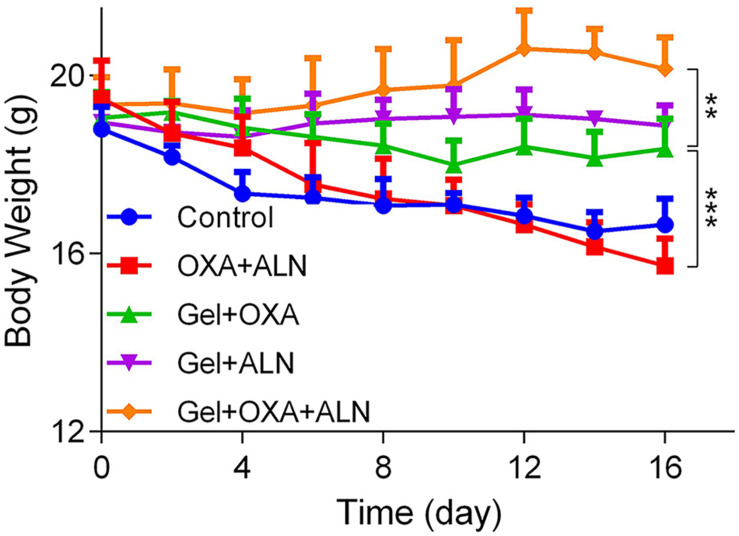
Body weight curve of each treatment group, the data is expressed as mean ± SD (*n* = 4, **P < 0.01, ***P < 0.001).

**FIGURE 7:**
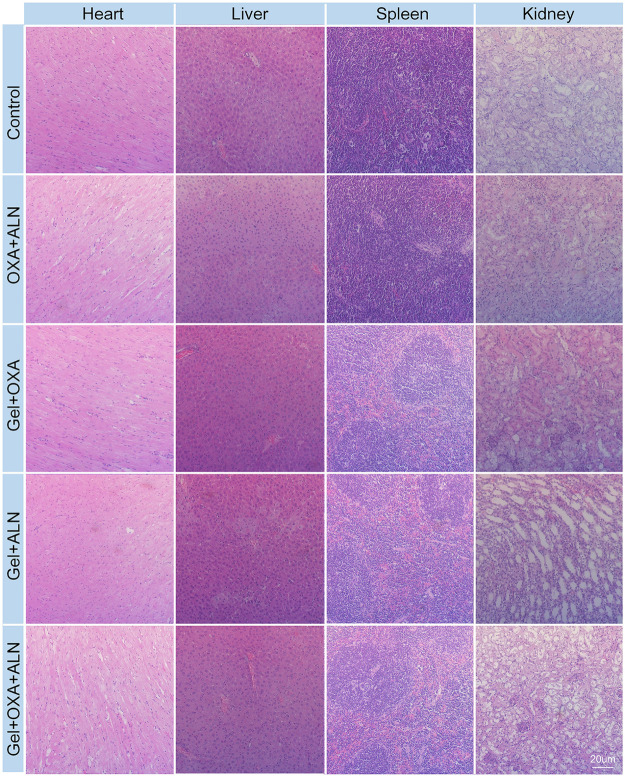
Histopathological analysis of organs in each treatment group.

Safety is a critical evaluation index for drug delivery systems. We verified the safety of Gel + OXA + ALN through weight monitoring and histopathological examination of several susceptible organs. As shown in Figure 6, the bodyweight of the mice in the control group showed a significant decrease because of no treatment. The malignant transformation of the tumor is very serious, and the tumor seriously damages the health of the mice. Due to the toxicity of free drugs, the OXA + ALN group had the most obvious weight-loss trend. The Gel + OXA and Gel + ALN groups showed different degrees of weight loss. The Gel + OXA + ALN group showed the best stable body weight and a slight weight increase. The H&E staining results of the main organs (heart, liver, spleen, kidney) could also indicate the toxicity of the long-term application of various therapeutic drugs to the body. As shown in Figure 7, we found that there were no noticeable morphological changes in the heart, liver, spleen and kidney of the tumor-bearing mice in the Gel + OXA + ALN group compared with the control group. The above results conclusively prove the low toxicity of Gel + OXA + ALN to all major organs in the whole body, indicating that it has great potential for clinical application.

## Conclusion

In this study, a system of synergistic *in situ* treatment of osteosarcoma with mPEG_45_–PLV_19_ thermosensitive hydrogel loaded with OXA and ALN was established. OXA can improve the immunogenicity of tumor and promote the accumulation of cytotoxic T cells, ALN can synergistically diminish tumors and has a bone-targeting effect to relieve bone destruction. The synergistic effect of them increases the therapeutic effect of this system on OS. The Gel + OXA + ALN group showed the highest anti-tumor activity and the most reliable safety *in vivo* in the mouse *in situ* OS model. The effective combination of mPEG_45_–PLV_19_ thermo-sensitive hydrogel with OXA and ALN provides broad prospects for *in situ* anti-osteosarcoma treatment.

## Data Availability Statement

All datasets generated for this study are included in the article/supplementary material.

## Ethics Statement

The animal study was reviewed and approved by the Animal Care and Use Committee at the Jilin University.

## Author Contributions

YS, CL, YZ, and DZ proposed and designed the experiments. YS, KL, and DZ carried out the experiments with the help of CL and YZ. YS, KL, and DZ drafted the manuscript and interpreted the data. CL and YZ revised the manuscript. All authors contributed to the article and approved the submitted version.

## Conflict of Interest

The authors declare that the research was conducted in the absence of any commercial or financial relationships that could be construed as a potential conflict of interest.
